# Dynamic Weighting Network for Person Re-Identification

**DOI:** 10.3390/s23125579

**Published:** 2023-06-14

**Authors:** Guang Li, Peng Liu, Xiaofan Cao, Chunguang Liu

**Affiliations:** 1 School of Control and Computer Engineering, North China Electric Power University, Beijing 102206, China; gl676484@gmail.com (G.L.);; 2 Yangzhong Intelligent Electric Research Center, North China Electric Power University, Yangzhong 212211, China; 3 School of Electronic and Information Engineering, Changchun University of Science and Technology, Changchun 130012, China

**Keywords:** re-identification, self-attention, fine-grained features

## Abstract

Recently, hybrid Convolution-Transformer architectures have become popular due to their ability to capture both local and global image features and the advantage of lower computational cost over pure Transformer models. However, directly embedding a Transformer can result in the loss of convolution-based features, particularly fine-grained features. Therefore, using these architectures as the backbone of a re-identification task is not an effective approach. To address this challenge, we propose a feature fusion gate unit that dynamically adjusts the ratio of local and global features. The feature fusion gate unit fuses the convolution and self-attentive branches of the network with dynamic parameters based on the input information. This unit can be integrated into different layers or multiple residual blocks, which will have varying effects on the accuracy of the model. Using feature fusion gate units, we propose a simple and portable model called the dynamic weighting network or DWNet, which supports two backbones, ResNet and OSNet, called DWNet-R and DWNet-O, respectively. DWNet significantly improves re-identification performance over the original baseline, while maintaining reasonable computational consumption and number of parameters. Finally, our DWNet-R achieves an mAP of 87.53%, 79.18%, 50.03%, on the Market1501, DukeMTMC-reID, and MSMT17 datasets. Our DWNet-O achieves an mAP of 86.83%, 78.68%, 55.66%, on the Market1501, DukeMTMC-reID, and MSMT17 datasets.

## 1. Introduction

Person re-identification (ReID) is a critical aspect of intelligent video analytics, particularly in situations where facial recognition fails or due to poor camera quality. With the rapid advancements in AI technology, scholars are becoming increasingly interested in integrating AI into security-related applications. Given the limitations of standard surveillance cameras and the sub-optimal performance of AI technologies, such as facial recognition, researchers have conducted extensive studies on the utilization of ReID within public security intelligent monitoring systems. While CNN-based methods have dominated ReID research for a long time and have recently experienced significant advances [1,2,3,4,5], the representation of global contextual information, critical in sophisticated computer vision tasks, continues to be a challenge. Although CNNs are powerful in feature extraction related to local information, they often struggle to represent contextual information sufficiently.

The Transformer architecture has been the focus of considerable attention in recent years [6]. This interest can be attributed, at least in part, to the success of self-attention-based Transformers in natural language processing (NLP), inspiring scholars to explore their potential application to various computer vision tasks, including image classification, object detection, and semantic segmentation [7,8,9,10]. Self-attention-based Transformers have demonstrated exceptional capabilities of capturing long-distance dependencies, presenting an attractive alternative to CNNs. The vision Transformer (ViT) [7] and data-efficient image Transformers (DeiT) [9] are two models that have replaced the conventional CNN backbone with a pure Transformer. In ViT, input images are partitioned into non-overlapping patches, each assigned a unique token. These patches are then processed using self-attention-based Transformer blocks that capture global relations and extract features for classification. Although Transformer-based models such as ViT and DeiT have inspired considerable interest, their utility for high-precision images is limited, as their ability to extract local features is relatively weak, and their use requires significant computational power, thereby hindering adoption for computer vision tasks. As a result, researchers have been actively investigating methods to integrate the Transformer architecture with CNNs to leverage both their strengths [11,12]. Several studies have used the Transformer in the CNN backbone by direct embedding, which not only allows for a more comprehensive assimilation of features and information, but also allows for a lower computational consumption than a pure Transformer model. Examples of these studies are AA-ResNet [13] and BoTNet [14].

**Figure 1:**
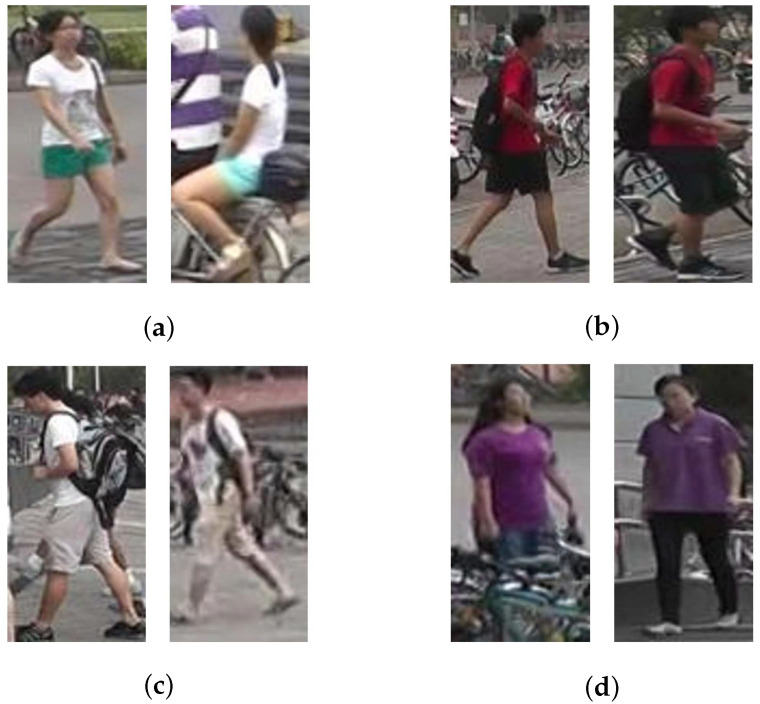
Each set of pictures in (**a**–**d**) contains two pictures of different people.

The ReID task is recognized for its intrinsic challenges, including subtle inter-class differences, significant intra-class variability, and heightened complexity relative to other computer vision tasks. In public spaces, individuals frequently wear similar clothing, bags, etc. (as depicted in Figure 1), resulting in necessitating comprehensive imformation encompassing long-distance feature dependencies and local features, especially fine-grained features. However, embedding long-distance dependencies by merely applying self-attention inevitably results in loss of fine-grained features, which are widely acknowledged to be crucial to the model’s performance. Consequently, developing specific modules that can balance the extraction of diverse features is indispensable for adapting the hybrid structure of CNNs and Transformers to the ReID task.

In this paper, we introduce a new ReID framework called DWNets, which can learn robust feature representations for ReID tasks. DWNets employs a parallel architecture that combines CNN-based local features and Transformer-based global features. Considering the differences between CNN and Transformer features, we added a convolutional activation module for the Transformer branch, containing 1 × 1 convolution and BatchNorm [15], LayerNorm [16] to balance these differences and facilitate feature fusion. We used a specially designed feature fusion gate (FFG) with dynamic weights to fuse CNN-style and Transformer-based features to reduce fine-grained feature loss.

Due to the specificity of the ReID task, multiple embeddings of long-distance dependencies are not appropriate. Hence, the ideal structure of the DWNet framework may vary depending on the CNN backbone used. We employed two representative backbones: ResNet [17] and OSNet [18]. In ResNet, we replaced the original CNN layer with a CNN-Transformer parallel structure in the fourth layer of the network. Conversely, in the lightweight network OSNet, we enhanced each residual block in the first layer of the network. Compared to the original model, our models achieved 2.5% and 2.2% mean average precision (mAP) improvements on the Market1501 dataset, while requiring minimal change in the number of parameters and computational consumption compared to the original model.

**Our contributions are summarized as follows:**

1. To enable the ReID model to retain the powerful ability to extract local features of CNNs while also acquiring long-distance dependencies without exceeding resource consumption limits, we conducted extensive experiments to investigate the feasibility and challenges of using a neural network model with a parallel structure of both CNNs and Transformers in the ReID task;
2. We propose the FFG to iteratively fuse CNN-based local features with Transformer-based global representations based on the problems identified in the experimental results. We experimentally verified the general applicability of the FFG;
3. We propose a high-performance ReID framework called DWNet, which is based on FFG. DWNet has an ability to fuse local features and global representations based on specific conditions. It outperforms the original baseline in the ReID task with comparable parameter complexity and computational consumption, demonstrating its potential to be the backbone of the ReID model.

## 2. Related Work

### 2.1. Object ReID

ReID is mainly aimed at person ReID and vehicle ReID. ReID can be viewed as a specialized object classification task. CNN structures have been the state-of-the-art approach in ReID for some time. A primary focus in ReID research is designing an appropriate loss function for training a CNN backbone on ReID tasks. Among many loss functions, triple loss [19] and cross-entropy loss (ID loss) [20] have gained significant popularity in ReID research. The ability to learn discriminative features is vital for enhancing ReID models, particularly for single images. Different from the object classification task, ReID task usually has some problems that the object classification task does not. For example, most pedestrian images are rectangular regions cut out according to the results of pedestrian detection algorithms in different environments. The pedestrian image itself is affected by the environment and the performance of the detection algorithm, which may have the problem of dislocation and occlusion. Moreover, ReID tasks have common attributes between classes and great intra-class differences. There is a lot of work to solve the re-ID task and design some other methods, which has gradually promoted the development of pedestrian re-ID. Some methods use human part detection to solve problems such as dislocation, e.g., ref. [21], dividing the human body into multiple parts, calculating the feature representation of these parts, and then computing the similarity of these representations. Ref. [22] proposed a dual-stream network model based on body part alignment. This model can represent the human posture as part of the feature map, and combine it directly with the overall appearance feature map to calculate the aligned pedestrian part representation. Some methods employ feature fusion to improve local feature learning. PCB [2] adopts a simple uniform division strategy that is more flexible than methods that require semantic segmentation. MGN [23] divides the entire network into a global feature representation branch and two local feature branches to extract multi-granularity features.

### 2.2. Transformer in Vision

Transformer’s effective capture of long-distance relationships is attributed to the use of multi-head self-attention (MHSA). Due to the success of Transformer in NLP, pure Transformer models have become increasingly popular. In recent years, several computer vision models have been developed for various tasks, including ViT [7] for image classification. ViT divides the input image into patches to mimic the sequence in NLP to enable the usage of Transformer. Owing to its exceptional performance, some subsequent ReID methods leverage the ViT backbone [24,25]. Nonetheless, the computational consumption of pure Transformer ReID models is prohibitively high, making them inapplicable in various scenarios.

## 3. Methods

With the powerful local feature extraction capability of CNN, the CNN-based model achieves higher accuracy at a lower cost, boosting the rapid development of computer vision. However, it focuses on aggregating local features, which hinders its capacity to acquire global representations, a limitation inherent to its structure. Although several techniques have been developed to overcome this challenge, they are restricted by their own structural problems and thus fall short of providing significant improvement. On the other hand, the Transformer-based model has an innate ability to capture global representations, thanks to the self-attention mechanism that enables it to capture long-distance relationships of sequences. Integrating CNN and Transformer network structures to enhance model performance in ReID tasks presents a challenging problem.

Drawing on [11,12,14], we attempt to implement a CNN and Transformer hybrid architecture that does not considerably increase computational demands while enhancing accuracy in the ReID task. Directly integrating a Transformer into a CNN leads to fine-grained features loss. To resolve this issue, we propose a parallel network structure called DWNet. Given that Transformer-based neural networks require extensive computations, we employ CNN as the foundation of the DWNet framework.

**Figure 2:**
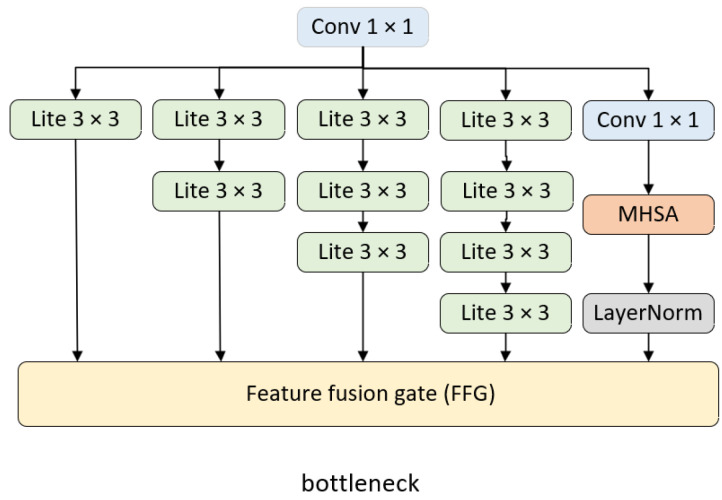
Bottlenecks in conv1 of DWNet-O.

**Figure 3:**
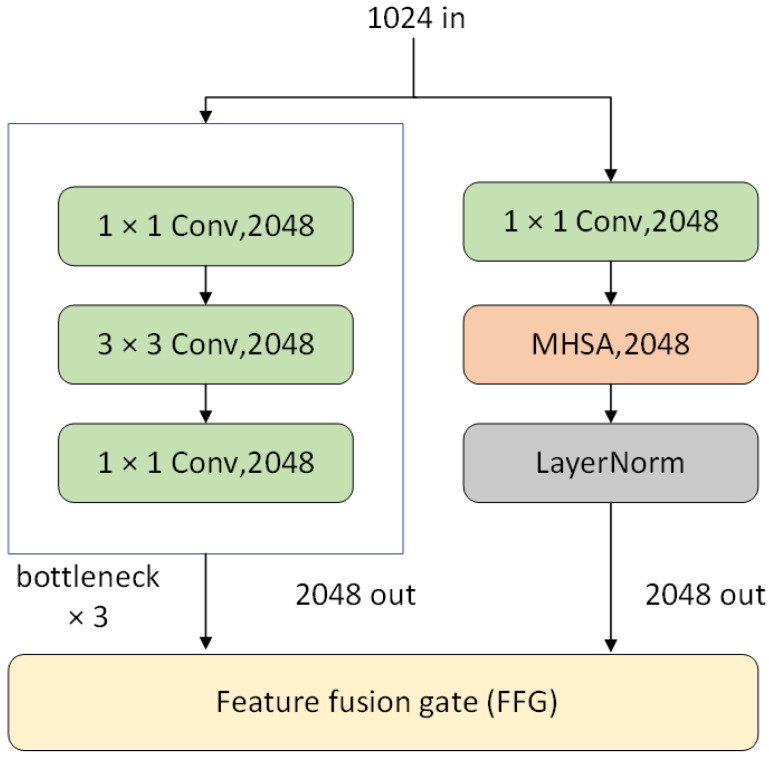
Layer 4 of DWNet-R.

DWNet’s primary concept is to utilize a parallel-merge structure by including CNN and Transformer branches for the fusion of local features and global representations. An essential aspect is a custom mechanism that dynamically adjusts the channel weights of the branches to minimize multiscale feature loss during branch merging. There are two main structures of DWNet. The first employs MHSA and FFG directly in the residual blocks, single-branch CNN, and multi-branch CNN, exhibited in Figure 2, where the network structure is adjustable by tuning the number of these residual blocks. The second replaces a specific layer of the original CNN with CNN and Transformer in parallel, using FFG in the connected layer as illustrated in Figure 3.

Based on our experiments, we have concluded that incorporating the self-attention mechanism multiple times to embed global long-distance feature dependency is often less effective than using it only once. This is especially true when it is overused. We have determined that while FFG within a residual block or stage can achieve a local optimum through adjustment of the weight parameter, using multiple residual blocks or stages to achieve a local optimum does not guarantee a global optimum. Our experimental results have enabled us to create the most effective DWNet structure for different CNN backbones, including two representative backbones—ResNet and OSNet—for the ReID task.

### 3.1. Feature Fusion Gate

**Figure 4:**
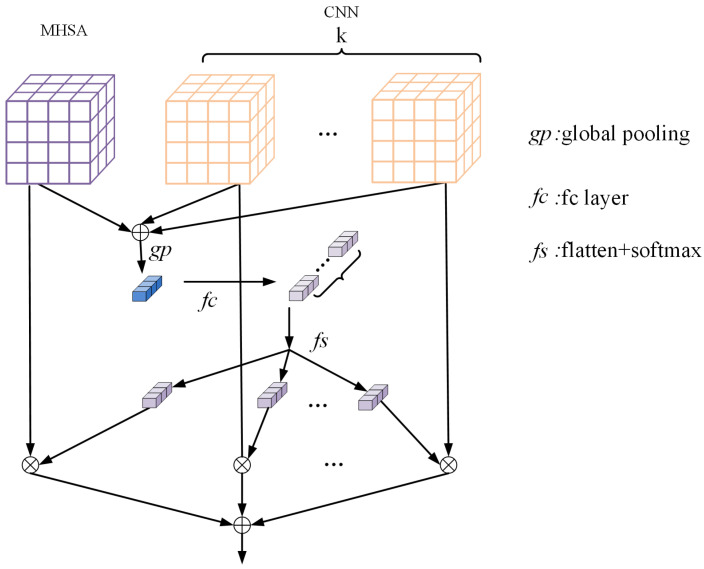
Structure of feature fusion gate.

There is misalignment [12] between the feature maps of the CNN branch and the output of the Transformer branch. Moreover, the simple connection is not well suited to the ReID task and will inevitably cause loss of fine-grained feature information in the CNN branch. To solve it, we propose for the FFG to adjust the weight of the feature map of the CNN and Transformer branches according to stimulus content, and then consecutively couple CNN-based local features with Transformer-based global representations by summing the feature map of the CNN branch and Transformer branch according to this weight. As illustrated in Figure 4, where the whole process is shown.

***Double branch*****:** For the given feature map of the CNN branch $X\in R^{H\times W\times C}$ and the given feature map of the multi-head self-attention branch $X\in R^{H^{\prime }\times W^{\prime }\times C^{\prime }},$ we conduct two transformations $\tilde{F}:\tilde{X}\to \tilde{U}\in R^{H\times W\times C}$ and $\hat{F}:\hat{X}\to \hat{U}\in R^{H^{\prime }\times W^{\prime }\times C^{\prime }}$ with the CNN branch and the MHSA branch, respectively. Note that $\tilde{F}$ and $\hat{F}$ have different compositions, where $\tilde{F}$ consists of efficient convolution, BatchNorm [15], and ReLU [26] in sequence. $\hat{F}$ consists of tuned convolution, MHSA, and activation layer in sequence.

***Multi-stream*****:** Some CNN residual blocks contain multiple streams, and to bring in the information of each stream, we use a new dimensional index k that denotes the number of CNN residual block streams. $\tilde{U}$ is the sum of increments of representations up to k:

(1) $$\begin{array}{cccc} & & {\tilde{U}}^{k}=\sum_{1}^{k}U_{j}^{k} & \end{array}$$

When *k* = 1, the CNN residual block consists of the convolution of a single stream, and when *k* > 1, the CNN residual block consists of multiple streams, each consisting of the convolution of the same or different kernel size.

***Calculate the weights*****:** First, we integrate information from each branch through summation. Then, we obtain global information by using global average pooling to generate channel-wise statistics. Specifically, we calculated the element of each channel and reshape the dimension from (h,w,c) to (s,c) by shrinking U through spatial dimensions H × W. We use a channel-wise parameter $z_{c}\in R^{C}$ to represent it

(2) $$\begin{array}{cccc} & & z_{c}=\frac{1}{H\times W}\sum_{i=1}^{H}\sum_{j=1}^{W}{\tilde{U}}_{c}^{k}\left(i,j\right)+\frac{1}{H\times W}\sum_{i=1}^{H}\sum_{j=1}^{W}{\hat{U}}_{c}\left(i,j\right) & \end{array}$$

Further, we set $\hat{z}$ to represent the result of the transformation. This is achieved by the full connected layer, and we use two convolution operations to reduce the dimensions for efficiency. The transformation is formulated as follows:

(3) $$\begin{array}{cccc} & & \hat{z}=T_{2}\left(\mathrm{rel}\left(B\left(T_{1}\left(z_{c}\right)\right)\right)\right) & \end{array}$$

Here, $T_{1}$ and $T_{2}$ are two convolution transformations, and rel is the ReLU function [26], B denotes the Batch Normalization [15] that can be learned to capture the importance of each channel. We use r to denote the dimensionality reduction multiplier, and the actual number of channels for Batch Normalization and ReLU function d=C/r. Then, we flatten $\hat{z}$ to (k + 1) dimensions for the next soft attention operation. The dimension of z is (k + 1,s,c)

(4) $$\begin{array}{cccc} & & z=F(\hat{z}) & \end{array}$$

For ease of expression, we will not distinguish between CNN streams and Transformer streams. We use $U_{i},i=\left(1,2,3,\ldots ,k+1\right)$ for each stream, where the first k streams are CNN streams and the last stream is a Transformer stream.

(5) $$\begin{array}{cccc} & & a_{i}\left(c\right)=\frac{1}{1+\exp\left(-M_{i}^{c}\left(z\right)\right)}\;\;i=\left(1,2,3,\ldots ,k+1\right) & \end{array}$$

Mapping $M_{i}^{c}$ determines the weight of each stream for the *c*-th channel based on z.

***Fuse*****:** The final feature map *V* is obtained by passing the soft attention weights for each stream. To facilitate the distinction between the streams of CNN and Transformer, we show the first *k* streams (CNN) and the last streams (Transformer) in Equation (6).

(6) $$V_{j}=\sum_{i=1}^{k}a_{i}\left(c\right)U_{i}+a_{k+1}\left(c\right)U_{k+1},\;\sum_{i=1}^{k+1}a_{i}\left(c\right)=1$$

(7) $$\begin{array}{cccc} & & V=\sum_{j=1}^{c}V_{j} & \end{array}$$

The output of the final feature fusion is $V\in R^{C\times H\times W}$.

### 3.2. DWNet Uses ResNet as the CNN Backbone (DWNet-R)

The DWNet-R model, which employs ResNet as the backbone, is composed of four parts. The first part is the CNN backbone, the second part is the CNN branch, the third part is the Transformer branch, and the fourth part is the FFG that connects these two branches. The entire model is referred to as DWNet-R, and layer four of DWNet-R can be observed in Figure 3. The stem component of DWNet is similar to ResNet, and both utilize the feature pyramid structure. The benefit of this structure is that the size of the feature map is reduced while the number of channels increases with each layer, thereby enhancing the feature extraction capability. Taking a cue from the ResNet50 structure, the entire structure can be divided into four layers. The first layer applies a 7 × 7 convolution and max pool technique, while the second through fourth layers comprise a varying number of bottlenecks, each of which contains two 1 × 1 convolutions to reduce computation and regulate the number of channels, and a 3 × 3 convolution. Finally, the output of each bottleneck is added to the input as a residual connection.

CNN Branch:

The CNN branch is consistent with the fifth layer of ResNet50 and consists of several bottlenecks (three in ResNet50).

Transformer Branch:

We use the multi-headed attention mechanism directly in the Transformer block of our DWNet model instead of in a separate component like ViT [7]. This block comprises a multi-head self-attention module, a down-projection fc layer, an up-projection fc layer, as well as LayerNorms that are implemented before each layer of both the fc layers and the self-attention module. In addition, we consider that the 3 × 3 convolution of the CNN branch has the ability to extract spatial location information and local features [27], which is similar to the position embedding technique employed in ViT, so we do not use the position embedding technique employed in ViT on the Transformer branch for the sake of streamlining the model.

### 3.3. DWNet Uses OSNet as the CNN Backbone (DWNet-O)

OSNet is an omni-scale feature learning network explicitly designed for the re-ID task. Similar to ResNet, OSNet includes multiple residual blocks, the exceptional attribute of which is their ability to capture features at various scales using multiple convolutional streams. To dynamically fuse the multi-scale features, OSNet introduces an aggregation gate.

OSNet leverages convolution operations of different core sizes to obtain features of various scales, including the use of multiple stacked 3 × 3 convolutions to perform 5 × 5 convolution operations. This powerful multi-scale feature extraction capability of OSNet allows us to achieve better performance on ReID tasks at a lower cost.

We directly apply the multi-head self-attention and FFG in the residual blocks, removing the unified aggregation gate. The original multi-scale convolution is concatenated to the FFG in the form of multiple branches and MHSA. To improve the model’s performance, we replaced the residual blocks in the first layer of the middle three layers of the original OSNet (which comprises two residual blocks per layer) with new residual blocks.

The resulting model is referred to as DWNet-O, whose conv1 is depicted in Figure 2.

## 4. Experiments

### 4.1. Datasets and Evaluation Protocol

We conducted extensive experiments on four datasets that have gained wide recognition in the ReID community: Market1501 [28], DukeMTMC-reID [29], MSMT17 [30], and CUHK03 [31]. Each of these four datasets has photos of each person at different angles and positions from different cameras.

We followed the criteria widely accepted by the Re-ID community for the experimental setup. We use standard metrics evaluated in the literature [28], including mean average precision (mAP) and cumulative matching characteristics (CMC) curves. In CMC, rank-n denotes the hit rate for ranking the first n results containing the correct labels. mAP is the average AP value calculated for all images. mAP is the average precision, i.e., the precision is averaged over the first n returned results for only those positions that return correct results. All of our experiments were run on specific cloud servers to flexibly tune the performance parameters, and we used a single NVIDIA RTX 3080Ti for training. The code toolbox used FastReID [32], and we modified the model code based on the project to accomplish our experimental purposes. We followed, to a large extent, the project’s configuration of the model and training parameters. In the training of the model, the images of each dataset are uniformly set to 256 × 128, the batch size is set to 128, and the model is trained for a total of 120 epochs. At the beginning, we set the basic learning rate to 3.5 × $10^{-4}$, which decreases as the training proceeds, and finally stabilizes at 3.5 × $10^{-6}$.

### 4.2. Ablation Experiments to Verify the Effectiveness of FFG

**Figure 5:**
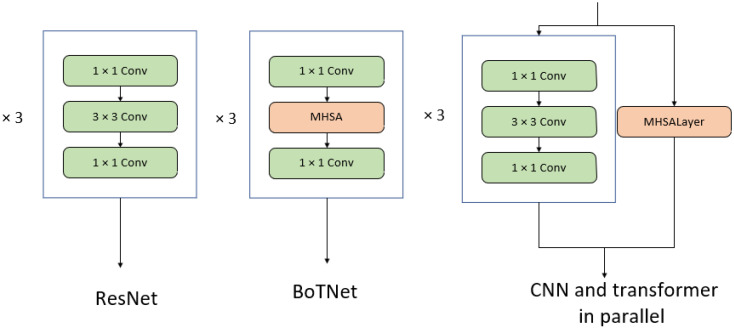
Comparison of Layer4 for different models.

BoTNet [14] is a simple, effective neural network model that embeds long-distance dependencies by replacing the convolution of a layer of ResNet with multi-headed self-attentiveness. BoTNet [14] obtained better results than the benchmark model ResNet in the instance segmentation task and image classification task. We first use experiments at the fourth layer of ResNet to verify the effectiveness of FFG. We used two network structures, BoTNet and convolutional and multi-head self-attention parallel networks, for comparison experiments with FFG. The comparison of the layer4 structure of each model is shown in Figure 5 and Table 1.

### 4.3. Ablation Experiments for Structure Selection of DWNet Framework

**DWNet uses ResNet as the Backbone (DWNet-R):**

ResNet uses varying numbers of bottlenecks in each of its four convolutional layers (layer1, layer2, layer3, layer4). In the previous section, we noted that the DWNet framework has two forms of embedding the Transformer, making it crucial to choose one and where to apply it. Consequently, we designed a series of ablation experiments. The experiments are divided into two parts. The first part involved embedding the Transformer directly into the residual block. On the other hand, the second part involved replacing the layers of ResNet with parallel layers of CNNs and Transformers. When using ResNet as the backbone, embedding the Transformer into each bottleneck of ResNet’s layers increases computational consumption, which is counterproductive to the study’s objective. Therefore, we only conducted experiments where we replaced each ResNet layer with parallel CNN and Transformer layers. However, experiments were only performed on the third and fourth layers because replacing the first two layers would significantly increase computational consumption, as shown in Table 2.

**DWNet uses OSNet as the Backbone (DWNet-O):**

The experiment with OSNet as the backbone is divided into two parts, the first part is to embed the Transformer directly in the residual block. We use [0,0,0] to denote the baseline OSNet, [1,0,0] to denote the bottleneck in the conv1 of OSNet embedded with the Transformer’s bottleneck replaced, and so on, [0,1,0], [0,0,1], [0,0,1] indicate that the bottlenecks in layer2, layer3, layer4 are replaced by the bottlenecks embedded in the Transformer. In addition, in another experiment, more than one layer of bottlenecks in OSNet were replaced. Where [1,1,0] represents the first and second layer bottlenecks in OSNet being replaced, with [0,1,1], [1,0,1], and [1,1,1] following the same pattern, As show in Table 3, Bold indicates that the number is the highest in the same column. Among them, the model with only the first layer of the bottleneck replaced has the best combined effect.

The second part is to directly replace the layers of OSNet with CNN and Transformer parallel layers. However, in contrast to ResNet, OSNet using this approach to embed the Transformer would significantly increase the computational consumption and is likely to be impossible to perform experiments under the given experimental conditions, so we have only selected structures that we can experiment on under the given conditions. We only selected the third layer for our experiments and obtained 84.87% mAP on the Martket1501 dataset, which is lower than the baseline, 75.14% mAP on the DukeMTMC-reID dataset, which is higher than the baseline, and 56.88% mAP on the MSMT17 dataset, which is not only higher than the baseline but also outperforms all the structures that replaced the bottleneck in the previous step better.

Taking into account the energy consumption and accuracy in each data set, we chose to replace the structure of the first layer of OSNet bottleneck with a replacement bottleneck as DWNet-O.

### 4.4. Comparison with Baseline and Other Methods

First, As shown in Table 4, we compare our models with the baseline models, taking Market1501 dataset as an example, DWNet-R is 2.20% higher than the baseline model in mAP and DWNet-O is 1.93% higher than the baseline model in mAP. Table 5 shows that DWNet achieves similar or even better accuracy compared to most classical pedestrian re-identification models. However, it still cannot reach the level of some recent excellent models, such as SCAL [33]. This is because we only improve the model’s performance by modifying the backbone to make it work well on devices with limited performance, while strictly limiting the model’s resource consumption. In contrast, other models do not have this limitation. As a result, our model achieves results comparable to most classical models, but there is still a gap compared to the best models. Compared with other models, we improve only on the backbone to improve the accuracy, which has better scalability and can be easily combined with other methods to further improve the model accuracy.

## 5. Discussion

### 5.1. Experimental Results and Analysis

**Figure 6:**
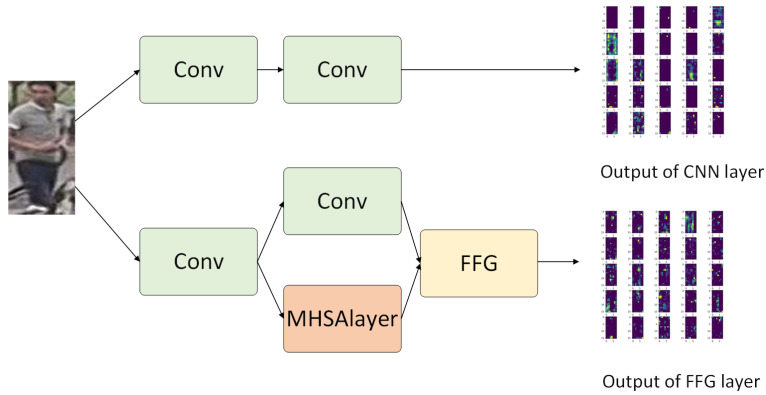
Comparison between using and not using FFG.

**Figure 7:**
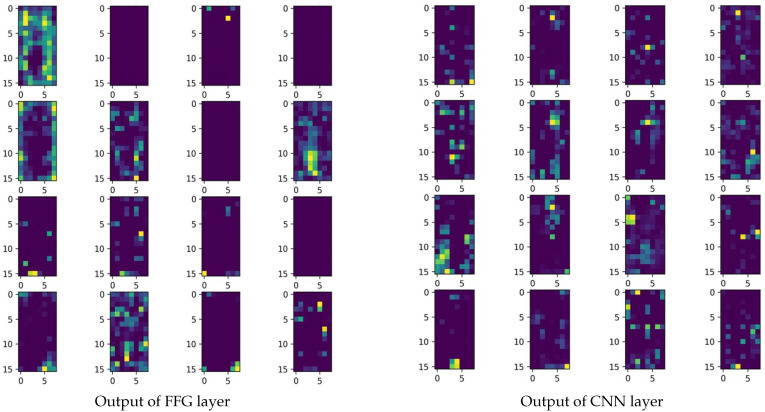
Two types of structural networks used to output feature maps based on the original image, followed by 16 randomly selected feature maps from the feature maps for display.

ResNet incorporates a variable number of bottlenecks in each of its four convolutional layers. According to the experimental data in the second step, replacing bottlenecks in layer1, layer2, layer3, and layer4 of the original ResNet with a bottleneck incorporating the Transformer enhances model performance. However, embedding the Transformer multiple times—for instance, replacing the p bottleneck in layer1 and layer2 simultaneously—yields no better performance results than embedding the Transformer once. Figure 6 and Figure 7 shows the feature map output of using FFG to embed the Transformer. When using FFG to fuse the feature map, it can be clearly seen that using FFG to retain CNN-based feature maps is less than not using FFG (there are more feature maps with completely inactive and only partially activated points in FFG). Although FFG can better preserve long-distance feature dependencies, it will result in the preservation of fewer feature maps that contain local feature information, especially fine-grained feature information. This reduces the ability to extract local features in the output of the next layer, resulting in the loss of local feature information. Embedding the Transformer multiple times worsens this problem, so its multiple embeddings may perform slightly worse than using just one or two Transformer embeddings, although it is still better than no embedding.

The experimental results show that replacing the bottlenecks in the original OSNet in conv2, conv3, and conv4 with the bottleneck embedded in the Transformer can improve the performance of the model. We find that the effect is less pronounced the higher the layer replacement is performed, and even on the MSMT17 dataset, when the bottleneck in conv4 is replaced, the performance improvement of the model is minimal in comparison. Embedding the Transformer at higher layers is due to the smaller size of the mapped feature maps. The size of the feature maps output by conv2, conv3, conv4 are 64 × 32, 32 × 16, and 16 × 8, respectively. This indicates that the effect of extracting long-distance dependencies directly using multi-head self-attention is affected by the size of the input feature map, and the larger the size of the input feature map, the more information about long-distance feature dependencies that can be extracted. In addition, the effect of feature fusion gate (FFG) is related to the number of output feature maps in place. When the number of output feature maps is higher, the more feature maps that can retain different local features, and the lower the loss of local features, especially fine-grained features, due to embedded long-distance feature dependencies. These two points lead to the fact that the optimal location for using FFG is greatly influenced by the network structure of backbone, for example, the optimal location for using FFG in ResNet and OSNet is very different, and sufficient experiments need to be conducted on exactly where to use FFG.

In the OSNet ablation experiments, we found that the approach of replacing the original layers with layers parallel to CNN and Transformer, while performing poorly on the Market1501 and DukeMTMC-reID datasets, performed well on the MSMT17 dataset, outperforming all the approaches of replacing bottlenecks. This shows that the optimal DWNet structure for each CNN backbone can not be generalized and needs to be combined with specific real-world situations.

As show in Table 6, we compare DWNet-R and DWNet-O with their original baselines, respectively. It can be seen that compared to the original baseline ResNet, DWNet-R slightly increases in the number of parameters and Flops, which is still within the acceptable range, and DWNet-O has almost no increase in the number of parameters and Flops, compared to the original baseline OSNet-O. We can find that DWNet is at a reasonable level in terms of the number of parameters, flops, and memory consumption, especially when applied to lightweight models—the increase in number of parameters and flops is less. The above work shows that DWNet is simple, efficient, and flexible. Compared with other ReID models that use Transformer, it has more advantages in terms of number of parameters, flops, and memory usage.

### 5.2. Ethical Considerations and Future Improvements for DWNet

First and foremost, we believe that moral and ethical considerations are paramount when dealing with aspects such as identification and data storage. Therefore, while developing the DWNet technology, we ensured that the datasets used were open-source, ethical, and free of legal issues. During our experiments, we complied with relevant ethical principles to ensure the security and privacy of the data and to prevent leakage of personal information. We also comply with relevant laws and regulations to ensure that our technology meets ethical and legal standards.

In practical security surveillance applications, models are often deployed on embedded devices. Given the performance constraints of these devices, backbone models such as ResNet and OSNet are typically used for pedestrian re-identification. DWNet, which offers comparable performance with reduced computational demands, can replace these backbone models without significantly increasing resource consumption while improving recognition accuracy. Furthermore, due to the high flexibility of DWNet, its structure can be adjusted to accommodate different environments. For instance, the DWNet structure that replaces the original layers of OSNet outperforms other structures on the MSMT17 dataset, which has a higher resolution than other datasets used in our experiments. As such, the DWNet structure can be employed in high-resolution camera scenes to enhance recognition rates.

In the future, we will continue to study the DWNet model structure to solve the problem that its dynamic weight parameters can only reach local optima but not global optima. This leads to a decrease in accuracy after embedding the transformer several times, as mentioned in our paper. We hope to make the dynamic weight parameters of DWNet globally optimal by increasing the losses at different stages so that embedding any number of transformers will only improve the accuracy of the model without degrading it.

## 6. Conclusions

The pure Transformer visual backbone architecture is computation intensive, so using CNN combined with self-attention visual backbone architecture, has become a popular field of research. To address the issues inherent in applying CNN combined with self-attention visual backbone architecture to the re-identification task, we propose a parallel framework based on feature fusion gates (FFG) for CNN combined with self-attention, called DWNet. Through ablation experiments, we demonstrate the general effectiveness of DWNet, and determined that different structures, DWNet-R and DWNet-O, improved performance compared to the original baseline while remaining computationally efficient. DWNet is simple, efficient, portable, and well-suited for large-scale industrial application scenarios. DWNet has the potential to serve as a backbone for re-identification tasks and can be easily combined with other methods to further improve the model accuracy.

## Figures and Tables

**Table 1:** The ablation study of FFG.

Model	Market1501		DukeMTMC-reID		MSMT17		CUHK03-L		CUHK03-D	
	mAP	rank-1	mAP	rank-1	mAP	rank-1	mAP	rank-1	mAP	rank-1
BoTNet-50	80.98	92.04	71.28	83.38	47.05	71.48	65.43	69.38	62.28	65.82
ResNet-50	85.23	94.01	76.33	84.85	48.80	74.26	68.28	69.88	67.05	67.96
CNN and Transformer in parallel	84.96	93.98	76.27	84.45	48.18	70.71	69.23	70.45	65.12	66.13
FFG	87.53	94.98	79.18	88.48	50.03	75.36	70.38	72.98	68.43	70.92

**Table 2:** The Ablation Study of DWNet-R.

Model	Market1501		DukeMTMC-reID		MSMT17		CUHK03-L		CUHK03-D	
	mAP	rank-1	mAP	rank-1	mAP	rank-1	mAP	rank-1	mAP	rank-1
Replaces the third layer	85.68	94.30	77.01	85.86	48.60	72.00	68.18	70.01	66.82	69.01
Replaces the fourth layer	87.53	94.98	79.18	88.48	50.03	75.36	70.38	72.98	68.43	70.92
ResNet-50	85.23	94.01	76.33	84.85	48.80	74.26	67.28	69.01	65.23	68.32

**Table 3:** The ablation study of the DWNet-O, (1,0,0), represents that the first layer of OSNet was replaced.

Model	Market1501		DukeMTMC-reID		MSMT17		CUHK03-L		CUHK03-D	
	mAP	rank-1	mAP	rank-1	mAP	rank-1	mAP	rank-1	mAP	rank-1
(0,0,0)	84.9	94.8	73.5	88.6	52.9	78.7	-	-	67.8	72.3
(1,0,0)	**86.83**	**95.9**	**78.68**	89.10	**55.66**	**78.96**	**71.96**	**74.00**	**68.81**	71.25
(0,1,0)	86.16	93.94	77.36	88.55	54.47	78.58	70.78	73.25	68.52	71.05
(0,0,1)	86.27	94.39	77.48	**89.18**	54.42	**78.67**	71.02	73.78	68.63	71.11
(1,1,0)	86.31	94.45	76.92	88.02	53.51	77.74	71.13	73.84	68.72	71.24
(1,0,1)	86.15	94.63	77.02	87.93	53.43	77.61	70.38	72.98	68.43	70.92
(0,1,1)	86.38	94.54	77.19	87.75	54.00	78.28	71.22	73.86	68.77	**71.29**
(1,1,1)	86.28	94.21	76.96	88.38	53.01	77.67	71.16	73.88	68.66	71.01

**Table 4:** The results of comparing the performance of our method with baseline models on four generic datasets.

Model	Market1501		DukeMTMC-reID		MSMT17		CUHK03-L		CUHK03-D	
	mAP	rank-1	mAP	rank-1	mAP	rank-1	mAP	rank-1	mAP	rank-1
DWNet-R	87.53	94.98	79.18	88.48	50.03	75.36	70.38	72.98	68.43	70.92
ResNet	85.23	94.01	76.33	84.85	48.80	74.26	67.28	69.01	65.23	68.32
DWNet-O	86.83	95.9	78.68	89.10	55.66	78.96	71.96	74.00	68.81	71.25
OSNet	84.9	94.8	73.5	88.6	52.9	78.7	-	-	67.8	72.3

**Table 5:** The results of comparing the performance of our method with other methods on four generic datasets.

Model	Market1501		DukeMTMC-reID		MSMT17		CUHK03-L		CUHK03-D	
	mAP	rank-1	mAP	rank-1	mAP	rank-1	mAP	rank-1	mAP	rank-1
PCB [10]	81.6	93.8	69.2	83.3	40.4	68.2	-	-	57.5	63.7
AANet [34]	83.4	93.9	74.3	87.7	-	-	-	-	-	-
DGNet [35]	86.0	94.8	73.5	88.6	52.3	77.2	-	-	-	-
BDB [36]	86.7	95.3	76.0	89.0	-	-	71.7	73.6	69.3	72.8
OSNet [18]	84.9	94.8	73.5	88.6	52.9	78.7	-	-	67.8	72.3
MGN [23]	86.9	95.7	78.4	88.7	52.1	76.9	68.0	67.4	66.8	66.0
IANet [37]	83.1	94.4	73.4	87.1	46.8	75.5	-	-	-	-
SCAL [33]	89.3	95.8	79.6	89.0	-	-	72.3	74.8	68.6	71.1
DWNet-R	87.53	94.98	79.18	88.48	50.03	75.36	70.38	72.98	68.43	70.92
DWNet-O	86.83	95.9	78.68	89.10	55.66	78.96	71.96	74.00	68.81	71.25

**Table 6:** Comparison of model parameters between DWNet and baseline.

Model	Total Params	Total Memory	Total MAdd	Total Flops	Total MemR+W
ResNet	23,508,032	97.75 MB	8.14 GMAdd	4.08 GFlops	252.05 MB
DWNet-R	55,782,720	112.77 MB	16.2 GMAdd	8.11 GFlops	399.69 MB
OSNet	1,905,828	104.69 MB	1.99 GMAdd	1.0 GFlops	214.96 MB
DWNet-O	1,976,372	115.76 MB	2.19 GMAdd	1.11 GFlops	234.33 MB

## Data Availability

Not applicable.
